# Effect of novel natural feed additive containing *Averrhoa bilimbi* L. fruit filtrate, wheat bran, and *Saccharomyces cerevisiae* on growth performance and meat characteristics of broilers

**DOI:** 10.14202/vetworld.2021.3007-3014

**Published:** 2021-11-27

**Authors:** Sugiharto Sugiharto, Anugrah R. Pratama, Turrini Yudiarti, Tugay Ayaşan

**Affiliations:** 1 Department of Animal Science, Faculty of Animal and Agricultural Sciences, Universitas Diponegoro, Semarang, Central Java, Indonesia; 2 Department of Organic Farming Business Management, Kadirli Faculty of Applied Sciences, University of Korkut Ata, Osmaniye, Turkey

**Keywords:** *Averrhoa bilimbi* L. fruit filtrate, breast meat, broiler, natural feed additive, organic acid

## Abstract

**Background and Aim:**

In the post-antibiotic era, consumer demand for healthy and safe meats has prompted poultry producers to seek alternative effective feed additives. This study aimed to investigate the effects of a novel natural feed additive based on a mixture of *Averrhoa bilimbi* L. fruit filtrate, wheat bran, and *Saccharomyces cerevisiae* on the growth rate, internal organ weight, and breast meat characteristics of broilers.

**Materials and Methods:**

A total of 280 1-day-old chicks were divided into one control (CNTRL; feed without additives) and three treatment groups: NOV25, feed with 2.5 g/kg novel additive; NOV50, feed with 5.0 g/kg novel additive; and NOV100, feed with 10 g/kg novel additive. The body weight (BW), feed intake (FI), and feed conversion ratio (FCR) were measured weekly. On day 35, the chickens from each group were slaughtered, and their internal organs and breast meat samples were collected.

**Results:**

The BW of broilers in NOV100 was greater (p=0.016) than that in the other groups. The FCRs in the treatments groups were lower (p<0.001) than that in the control group. Elevated levels of the novel additive increased (p=0.051) the relative weight of the duodenum. The pH values in the breast meat of broilers receiving the novel additive were higher (p<0.001) than that in control. The C20:3n-6 of the NOV100 breast meat was lower (p=0.012) than that of NOV25 and NOV50, but it did not differ from that of the control. The unsaturated fatty acid-to-saturated fatty acid ratio in the breast meats of the treatments was higher (p=0.032) than that in control. The L-tyrosine content in NOV50 breast meat was higher (p=0.036) than that in CNTRL and NOV100 but did not differ from that in NOV25.

**Conclusion:**

The proposed feed additive improved the live BW and FCR of broilers and the physical and nutritional qualities of broiler breast meat.

## Introduction

The broiler industry has expanded steadily in Indonesia over the past 10 years, representing a substantial part of the national economy today [1]. To optimize broiler productivity and health in the post-antibiotic era, farmers commonly use feed additives. These additives include probiotics, prebiotics, synbiotics, organic acids, enzymes, fatty acids, and phytobiotics or plant-derived products [2]. Indonesia is known for its abundance of medicinal plants, which are used as additives and supplements for livestock in addition to being used for human medicine. *Averrhoa bilimbi* L. is one of several herbal plants with the potential to be used as an additive for broilers. The fruit filtrate of this plant is naturally acidic (high in citric acid) and can be used as a natural acidifier to increase broiler growth and improve health [3,4]. It contains lactic acid bacteria (LAB) [3,5], which function as probiotics for broiler chickens [4]. A previous study reported that using acidifiers and probiotics in combination had a greater impact on broiler production and health than using an acidifier and probiotic separately [2]. In addition, several studies have reported that combining probiotics with prebiotics increased probiotic efficacy compared with using probiotics alone [6,7]. Wheat bran has been identified as a prebiotic-rich and affordable feed ingredient for broiler chickens [8]. This by-product of the wheat milling industry is rich in arabinoxylan oligosaccharides, which function as an energy source for bacteria [9].

Consumers are becoming increasingly aware of the quality and health impacts of the meat they consume. This has prompted broiler farmers to produce healthier meats. Dietary supplementation of organic acids reduces the contents of saturated fatty acids (SFAs) and increases the polyunsaturated fatty acids (PUFAs) of broiler meat [10]. In another study, feeding LAB-based probiotics and *Saccharomyces cerevisiae* to broilers improved fatty acid profiles of the meat [11]. Similarly, feeding prebiotics increased the proportions of PUFA and n-3 PUFA in broiler meats [12]. We, therefore, hypothesized that combining acidifiers, probiotics, and prebiotics in a feed additive would improve the productive performance and meat quality of broiler chickens through synergistic effects. For this study, the fruit filtrate of *A. bilimbi* L. was used as an acidifier and source of probiotic LAB, and prebiotics were derived from wheat bran. To augment the probiotic effect, the yeast *S. cerevisiae* was added to the feed additive.

The aim of this study was to assess the effect of a novel natural feed additive based on a mixture of *A. bilimbi* L. fruit filtrate, wheat bran, and *S. cerevisiae* on growth rate, internal organ weight, and breast meat traits of broilers.

## Materials and Methods

### Ethical approval

The Committee of Animal Ethics of the Faculty of Animal and Agricultural Sciences, Universitas Diponegoro approved the *in vivo* experiment (No. 57-02/A3/KEP/FPP), which was carried out in conjunction with the standard animal husbandry and health guidelines outlined in Legislation of the Republic of Indonesia No. 18, 2009.

### Study period and location

The study was conducted from January to March 2021 at the Broiler Experimental House of the Faculty of Animal and Agricultural Sciences, Universitas Diponegoro, Semarang, Central Java, Indonesia.

### Production of feed additive

Ripe *A. bilimbi* L. fruits (identified by Department of Biology, the Faculty of Science and Mathematics, Universitas Diponegoro, Semarang) were gathered from the local campus gardens (Tembalang Campus, Semarang, Central Java, Indonesia), cleaned with water, and then crushed using a portable electric blender at medium speed. The fruit juice was filtered using a cheese cloth, and the obtained fruit filtrate (pH 1.98) was used to ferment the wheat bran. Before fermentation, the wheat bran was prepared according to Utama *et al*. [13]. Bran was added to distilled water at a ratio of 3:1 (g: mL); the solution was thoroughly mixed, autoclaved (electric All-American^®^ Sterilizer, Westbury, NY, USA) at 121°C for 15 min, and then left to cool down to room temperature (around 25°C). To produce the feed additive, the autoclaved wheat bran was mixed with the fruit filtrate at a ratio of 1:4 (g: mL) and incubated anaerobically using an anaerobic jar (Oxoid, Thermo Fisher Scientific, Waltham, MA, USA) at 38°C for 2 days. The fermented product was subsequently sun-dried and milled using an electric grinder (Panasonic Grinder MXGX1462, PT. Panasonic Gobel Indonesia, Jakarta, Indonesia). Finally, the fermented product was combined with commercial yeast *S. cerevisiae* at a ratio of 2:1 (g: g; Angel Yeast Co. Ltd., Hubei, China; contained 9.82×10^11^ colony forming unit [CFU]/g). The natural feed additive contained LAB of 5.47×10^11^ CFU/g (based on the total plate count method using de Man, Rogosa, and Sharpe agar, incubated at 38°C for 48 h [14]) with pH 4.65. Based on a standard proximate analysis, the feed additive contained 9.85% moisture, 14.86% crude protein, 0.30% crude fat, 1.66% fiber, and 8.72% ash (on a dry matter basis).

### *In vivo* experiment

For the experiment, 280 1-day-old chicks (unsexed Lohmann MB-202 broiler strain; average body weight [BW] of 48.2±0.26 g) were divided into four treatment groups with seven replications (each containing 10 chicks). These treatment groups included the control (CNTRL; feed without the additive), NOV25 (feed with 2.5 g/kg of the additive), NOV50 (feed with 5.0 g/kg of the additive), and NOV100 (feed with 10 g/kg of the additive). The birds were reared for 35 days in an opened broiler house in 1 m^2^ pens. The photoperiod was 24 h light. Plastic curtains and light bulbs were used to regulate the temperature and relative humidity in the broiler house. The temperature was 32°C for the first 4 days and 28-29°C for the remainder of the experiment. The relative humidity was maintained at approximately 80%.

Feeds were prepared in mash form following the Indonesian National Standards for Broiler Feed [15] as starter (days 1-21) and finisher (days 22-35) diets (Table-1) [16]. During the mixing process, the additives were added proportionately to the main feed according to the ratio of the specific treatment group. No antibiotics or other additives such as enzymes, phytase, amylase, carbohydrases, or coccidiostats were added. Feed and water were provided *ad libitum* in each pen using a manual feeder/drinker. The birds were immunized with Newcastle disease vaccine through eye drops and drinking water on days 4 and 18, respectively. On day 12, they were given the Gumboro (infectious bursal disease) vaccine through their drinking water.

**Table-1:** Ingredients and nutritional compositions of feeds.

Item (%, unless otherwise noticed)	Starter (days 1-21)	Finisher (days 22-35)
Yellow corn	53.50	61.00
Palm oil	2.320	2.950
Soybean meal, crude protein 44.15%	40.13	32.00
DL-methionine, 990 g	0.190	0.190
Bentonite	0.750	0.750
Limestone	1.000	1.000
Monocalcium phosphate	1.300	1.300
Premi×^1^	0.340	0.340
Chlorine chloride	0.070	0.070
Salt	0.400	0.400
Calculated chemical components		
ME, (kcal/kg)^2^	2,900	3,023
Crude protein	22.00	19.01
Crude fiber	5.470	5.530
Ca	1.140	1.110
P	0.570	0.580
Proximate components		
Moisture	10.00	10.59
Crude protein	19.00	18.75
Crude fat	3.170	5.270
Crude fiber	5.920	6.800
Ash	10.44	9.080

^1^Provided per kg of feed: 1100 mg Zn, 1000 mg Mn, 75 mg Cu, 850 mg Fe, 4 mg Se, 19 mg I, 6 mg Co, 1225 mg K, 1225 mg Mg, 1,250,000 IU Vitamin A, 250,000 IU Vitamin D_3_, 1350 g pantothenic acid, 1875 g Vitamin E, 250 g Vitamin K_3_, 250 g Vitamin B_1_, 750 g Vitamin B_2_, 500 g Vitamin B_6_, 2500 mg Vitamin B_12_, 5000 g niacin, 125 g folic acid, and 2500 mg biotin. ^2^Metabolizable energy was predicted based on formula [16]: 40.81 (0.87 [crude protein+2.25 crude fat+nitrogen-free extract]+2.5)

### Data collection and analysis

The live BW, amount of feed intake, and feed conversion ratio (FCR) of chicks were determined weekly. On day 35, one chick from each pen (seven chicks per treatment group) was slaughtered, defeathered, and dissected. The internal organs of the birds were collected and weighed (empty condition) using an analytical balance (Henherr, ACS-718, China). The carcass and commercial cuts (breast, wings, thigh, drumstick, and back) were also inspected. Likewise, the meat samples were collected from the breast to determine physical properties such as water-holding capacity (WHC) and pH, as well as fatty acid and amino acid profiles.

The WHC of breast meats was measured using the Grau-Hamm [17] method. The pH of the meat was measured using a portable pH meter (OHAUS ST300, Ohaus, Parsippany, NJ, USA). The concentrations of fatty acids in the breast meat samples were measured using a standard gas chromatography (Shimadzu Corporation, Kyoto, Japan) method. The presence of fatty acids was determined by comparing the retention times of each sample to the retention times of standard. Fatty acid quantification was performed by normalizing and converting the area percentage to g/100 g of the edible section using a lipid conversion factor [18]. The amino acid content of the breast meat samples was determined using a typical ultra-performance liquid chromatography protocol (Waters, Massachusetts, USA) according to the Waters Acquity UPLC H-Class and H-Class Bio Amino Acid Analysis System Guide [19]. The process employed a 1.7 m (2.1×100 mm) AccQ.Tag Ultra C18 column with a column temperature of 49°C, handheld phase flow speeds of 0.5 mL/min, 1 mL of injection capacity, and a photometric diode array (Waters) detector with the 260 nm wavelength. The mobile step composition schemes were as follows: Eluent A Amino Acid Analysis AccQ.Tag Ultra concentrate; Eluent B Amino Acid Analysis AccQ.Tag Ultra 10% in water; C: Distilled water; and D: Eluent B Amino Acid Analysis AccQ.Tag Ultra.

### Statistical analyses

Data were tested for normal distribution and homogeneity of variance and then analyzed using analysis of variance (ANOVA, Statistical Package for the Social Sciences 16.0 version, SPSS Inc., Chicago, IL, USA). For treatments with p<0.05, a Duncan multi-range test was performed. The influence of the different levels of additives in the treatments was assessed using linear regression. Pens/replicates were regarded as the experimental units. The normal distribution and homogeneity of variance were tested before ANOVA test. The tendency was considered when p>0.050 and p<0.100.

## Results

### Broiler performance

Data for BW, feed intake (FI), and FCR of the broilers are listed in Table-2. On days 21 and 35, the live BW of broilers in NOV100 was higher (p=0.026 and p=0.016, respectively) than that of the other groups. The cumulative FI in NOV50 was lower (p=0.038) than that in CTRL and NOV100, but it did not differ from that in NOV25 on day 35. The FCR in NOV100 was lower (p<0.001) than that in CNTRL and NOV25, but it did not differ from that in NOV50.

**Table-2:** Performances of broilers.

Item	Treatment				SEM	p-value	
	
	CNTRL	NOV25	NOV50	NOV100		A	L
Day 21							
BW (g)	618.8^b^	638.6^b^	637.2^b^	688.1^a^	8.804	0.026	0.006
Accumulative FI (g)	816.6	841.2	824.4	847.9	7.399	0.430	0.252
FCR	1.431	1.432	1.403	1.328	0.016	0.068	0.017
Day 35							
BW (g)	1689^b^	1710^b^	1673^b^	1826^a^	19.62	0.016	0.031
Accumulative FI (g)	2534^a^	2465^ab^	2361^b^	2550^a^	26.56	0.038	0.818
FCR	1.543^a^	1.486^b^	1.453^bc^	1.436^c^	0.011	<0.001	<0.001

^a,b,c^Means with various letters within the same row are substantially different (p<0.05). CNTRL=Chicks given feed without additives, NOV25=Chicks given feed administrated with 0.25% novel additive, NOV50=Chicks given feed administrated with 0.50% novel additive, NOV100=Chicks given feed supplemented with 1.00% novel additive, BW=Body weight, FI=Feed intake, FCR=Feed conversion ratio, A=Analysis of variance (ANOVA), L=Linear regression, SEM=Standard error of the means

### Internal organ weight and carcass yield of broilers

Table-3 depicts internal organ weight (relative to the live BW). ANOVA indicated no influence (p>0.050) of the dietary additive on the measured internal organ weight of broilers. However, the additive seemed to increase the weight of the duodenum.

**Table-3:** Relative weight of internal organ of broilers.

Item (% live BW)	Treatment				SEM	p-value	
	
	CNTRL	NOV25	NOV50	NOV100		A	L
Heart	0.501	0.526	0.503	0.491	0.011	0.722	0.584
Liver	2.103	2.071	2.286	2.157	0.048	0.422	0.388
Proventriculus	0.539	0.507	0.510	0.469	0.018	0.619	0.206
Gizzard	1.404	1.393	1.480	1.407	0.031	0.759	0.734
Pancreas	0.266	0.287	0.320	0.313	0.015	0.570	0.192
Duodenum	0.378	0.459	0.476	0.476	0.018	0.163	0.051
Jejunum	0.954	1.050	1.129	1.073	0.029	0.190	0.092
Ileum	0.714	0.843	0.910	0.761	0.033	0.167	0.496
Caeca	0.641	0.673	0.501	0.700	0.034	0.169	0.989
Abdominal fat	0.751	1.093	0.946	1.007	0.064	0.294	0.299
Spleen	0.103	0.127	0.114	0.127	0.001	0.853	0.728
Thymus	0.269	0.267	0.247	0.266	0.015	0.961	0.839
Bursa of Fabricius	0.160	0.183	0.173	0.160	0.011	0.880	0.988

CNTRL=Chicks given feed without additives, NOV25=Chicks given feed administrated with 0.25% novel additive, NOV50=Chicks given feed administrated with 0.50% novel additive, NOV100=Chicks given feed supplemented with 1.00% novel additive, BW=Body weight, A=Analysis of variance (ANOVA), L=Linear regression, SEM=Standard error of the means

Carcass weights and commercial proportions (Table-4) of chickens were not significantly affected by the treatments (p>0.050).

**Table-4:** Carcass and commercial proportions of broilers.

Item	Treatment				SEM	p-value	
	
	CNTRL	NOV25	NOV50	NOV100		A	L
Eviscerated carcass (% live BW)	70.17	69.06	65.69	60.00	2.530	0.470	0.120
		% eviscerated carcass				
Breast	37.17	38.01	36.35	33.37	1.457	0.689	0.301
Wings	10.66	10.46	10.23	9.048	0.453	0.578	0.191
Thigh	16.80	16.30	16.28	14.06	0.620	0.380	0.114
Drumstick	14.47	13.77	14.98	12.36	0.550	0.358	0.249
Back	20.89	21.46	22.16	18.76	0.822	0.501	0.383

CNTRL=Chicks given feed without additives, NOV25=Chicks given feed administrated with 0.25% novel additive, NOV50=Chicks given feed administrated with 0.50% novel additive, NOV100=Chicks given feed supplemented with 1.00% novel additive, BW=Body weight, A=Analysis of variance (ANOVA), L=Linear regression, SEM=Standard error of the means

### Physical and chemical characteristics of the meat samples

The breast meat pH values of the treatment groups were higher than that of the control, with NOV100 exhibiting the highest value. The WHC of the breast meat did not differ among all groups (Table-5).

**Table-5:** Water-holding capacity and pH values of broiler meats.

Item	Treatment				SEM	p-value	
	
	CNTRL	NOV25	NOV50	NOV100		A	L
WHC (%)	35.89	36.98	36.55	36.93	0.170	0.081	0.084
pH	6.817^c^	6.871^b^	6.877^b^	6.932^a^	0.009	<0.001	<0.001

^a,b^Means with various letters within the same row are substantially different (p<0.05). CNTRL=Chicks given feed without additives, NOV25=Chicks given feed administrated with 0.25% novel additive, NOV50=Chicks given feed administrated with 0.50% novel additive, NOV100=Chicks given feed supplemented with 1.00% novel additive, BW=Body weight, WHC=Water-holding capacity, A=Analysis of variance (ANOVA), L=Linear regression, SEM=Standard error of the means

Table-6 presents the fatty acid compositions of broiler breast meat samples. NOV100 chicks had a lower (p=0.012) proportion of dihomo-gamma-linolenic acid (C20:3n-6) than chicks from NOV25 and NOV50. However, the proportion did not differ significantly from that of the CNTRL birds. The unsaturated fatty acid (UFA)-to-SFA ratio in the breast meat samples of NOV25, NOV50, and NOV100 was higher (p=0.032) than that in CNTRL. Other fatty acids compositions did not vary significantly (p>0.050) among the groups.

**Table-6:** Fatty acid profiles of broiler meats.

Item (g/100 g)	Treatment				SEM	p-value	
	
	CNTRL	NOV25	NOV50	NOV100		A	L
Myristic acid (C14:0)	0.004	0.003	0.008	0.003	<0.001	0.155	0.921
Pentadecylic acid (C15:0)	ND	ND	ND	ND	NA	NA	NA
Palmitic acid (C16:0)	0.222	0.216	0.358	0.149	0.031	0.094	0.784
Stearic acid (C18:0)	0.084	0.082	0.117	0.053	0.009	0.072	0.481
Arachidic acid (C20:0)	ND	ND	ND	ND	NA	NA	NA
Heneicosanoic acid (C21:0)	ND	ND	ND	ND	NA	NA	NA
Behenic acid (C22:0)	ND	ND	ND	ND	NA	NA	NA
Tricosylic acid (C23:0)	ND	ND	ND	ND	NA	NA	NA
Lignoceric acid (C24:0)	ND	ND	ND	ND	NA	NA	NA
Myristoleic acid (C14:1n9c)	ND	ND	ND	ND	NA	NA	NA
Pentadecanoic acid (C15:1n9t)	ND	ND	ND	ND	NA	NA	NA
Palmitoleic acid (C16:1n7)	0.028	0.032	0.059	0.021	0.005	0.060	0.904
Oleic acid (C18:1n9c)	0.333	0.336	0.561	0.242	0.049	0.129	0.918
Elaidic acid (C18:1n9t)	ND	ND	ND	ND	NA	NA	NA
Eicosenoic acid (C20:1)	0.003	0.003	0.004	0.003	0.001	0.948	0.834
Gondoic acid (C20:1n9)	ND	ND	ND	ND	NA	NA	NA
Erucic acid (C22:1n9)	ND	ND	ND	ND	NA	NA	NA
Nervonic acid (C24:1n9)	ND	ND	ND	ND	NA	NA	NA
Linoleic acid (C18:2n-6c)	0.153	0.154	0.248	0.105	0.021	0.095	0.788
Linolelaidic acid (C18:2n-6t)	ND	ND	ND	ND	NA	NA	NA
Gamma-linolenic acid (C18:3n-6)	ND	ND	ND	ND	NA	NA	NA
Alpha-linolenic acid (C18:3n-3)	0.004	0.003	0.008	0.003	0.002	0.151	0.804
Eicosadienoic acid (C20:2n-6)	ND	ND	ND	ND	NA	NA	NA
Dihomo-gamma-linolenic acid (C20:3n-6)	0.008^ab^	0.009^a^	0.011^a^	0.004^b^	0.001	0.012	0.206
Eicosatrienoic acid (C20:3n-3)	ND	ND	ND	ND	NA	NA	NA
Arachidic acid (C20:4n-6)	0.028	0.034	0.036	0.018	0.003	0.110	0.255
Eicosapentaenoic acid, EPA (C20:5n-3)	ND	ND	ND	ND	NA	NA	NA
Docosadienoic acid (C22:2n-6)	ND	ND	ND	ND	NA	NA	NA
Docosapentaenoic acid (C22:5n-3)	ND	ND	ND	ND	NA	NA	NA
Docosahexaenoic acid, DHA (C22:6n-3)	ND	ND	ND	ND	NA	NA	NA
Total SFA	0.311	0.301	0.484	0.205	0.040	0.089	0.711
Total UFA	0.562	0.577	0.932	0.400	0.080	0.109	0.856
Total MUFA	0.365	0.371	0.625	0.276	0.056	0.123	0.936
Total PUFA	0.198	0.206	0.307	0.134	0.025	0.089	0.686
n-6 PUFA	0.190	0.197	0.295	0.128	0.023	0.089	0.686
n-3 PUFA	0.004	0.003	0.008	0.003	0.001	0.180	0.678
UFA: SFA	1.786^b^	1.912^a^	1.903^a^	1.924^a^	0.019	0.032	0.017
PUFA: SFA	0.632	0.693	0.657	0.669	0.014	0.506	0.566
(n-6):(n-3) PUFA	23.68	16.75	30.76	18.45	3.679	0.556	0.961

^a,^ ^b^Means with various letters within the same row are substantially different (p<0.05). CNTRL=Chicks given feed without additives, NOV25=Chicks given feed administrated with 0.25% novel additive, NOV50=Chicks given feed administrated with 0.50% novel additive, NOV100=Chicks given feed supplemented with 1.00% novel additive, SFA=Saturated fatty acid, UFA=Unsaturated fatty acid, MUFA=Monounsaturated fatty acid, PUFA=Polyunsaturated fatty acid, A=Analysis of variance (ANOVA), L=Linear regression, SEM=Standard error of the means. ND=Not detected, NA=Not statistically analyzed

Data on the amino acid profile of broiler meats are presented in Table-7. The NOV50 meats contained higher amino acid L-tyrosine than those of CNTRL and NOV100 (p=0.036), but they did not differ significantly from that of NOV25 meat. There was no variation (p>0.050) in amino acid contents of broiler meats among the treatment groups.

**Table-7:** Amino acid profiles of broiler meats.

Items (g/kg)	Treatments				SEM	p-value	
	
	CNTRL	NOV25	NOV50	NOV100		A	L
L-Histidine	10.92	9.317	10.75	8.321	0.947	0.758	0.464
L-Threonine	10.75	11.92	13.06	11.58	0.428	0.303	0.356
L-Proline	7.349	13.25	9.747	7.051	1.231	0.262	0.698
L-Tyrosine	7.756^b^	9.236^ab^	12.54^a^	7.846^b^	0.687	0.036	0.571
L-Leucine	15.49	18.73	16.41	17.52	1.081	0.761	0.703
L-Aspartate acid	20.76	17.10	19.37	22.54	0.948	0.225	0.381
L-Lysine	20.34	19.53	16.54	20.99	1.085	0.504	0.915
Glycine	10.23	9.511	10.89	9.241	0.435	0.558	0.688
L-Arginine	14.43	13.42	17.53	15.28	0.852	0.385	0.392
L-Alanine	12.80	14.00	12.68	13.80	0.442	0.647	0.678
L-Valine	12.04	13.39	14.75	11.51	1.027	0.705	0.979
L-Isoleucine	10.66	12.22	11.01	10.75	0.497	0.686	0.836
L-Phenylalanine	9.473	13.76	13.62	10.17	1.070	0.359	0.842
L-Glutamic acid	34.41	28.64	26.05	38.34	2.205	0.191	0.650
L-Serine	9.106	12.64	10.33	9.222	0.644	0.183	0.741

^a,b^Means with various letters within the same row are substantially different (p<0.05). CNTRL=Chicks given feed without additives, NOV25=Chicks given feed administrated with 0.25% novel additive, NOV50=Chicks given feed administrated with 0.50% novel additive, NOV100=Chicks given feed supplemented with 1.00% novel additive, A=Analysis of variance (ANOVA), L=Linear regression, SEM=Standard error of the means

## Discussion

Our results reveal that on days 21 and 35, dietary supplementation with the mixture of *A. bilimbi* L. fruit filtrate, wheat bran, and *S. cerevisiae* linearly increased the live BW and decreased the FCR of broilers with increasing levels of the additive. The most prominent effect was observed at a concentration of 10 g/kg of feed. This treatment (containing crude protein of 14.86%) increased the protein levels of the diet and consequently increased the broilers’ growth rate. In this study, additive supplementation increased the relative weight of the duodenum, which seemed to be associated with an improved intestinal morphology [20], and it, hence, improved the intestinal functions of broilers in digesting and absorbing the nutrients. This is suggested by the improved FCRs in the treatment groups. A study by Pratama *et al*. [4] reported that fermented *A. bilimbi* L. fruit filtrate increased the villi length of jejunum of broilers. Similarly, wheat bran [21] and *S. cerevisiae* [22] have been confirmed to improve the intestinal morphology of broilers.

Our findings reveal no impact of the dietary additive on the yield of carcass and commercial proportions. This is consistent with Pratama *et al*. [4] who reported no significant effect of fermented *A. bilimbi* L. fruit filtrate on the broilers’ carcass characteristics. Regarding wheat bran, Semjon *et al*. [23] reported no effect of fermented wheat bran on the carcass yield of broilers. Yalçın *et al*. [24] did not observe any impact of feeding *S. cerevisiae* on the yield. Regarding the pH value, high values are generally associated with a high WHC and high protein content in meats [25]. Our results reveal that the pH values of breast meat samples increased with increasing levels of feed additive and were correlated with increasing WHC. This study did not measure the protein content of breast meat. However, the literature suggests that dietary acidifiers increase protein digestibility, as well as protein biosynthesis, and reduce protein degradation in birds [26]. In addition, the dietary inclusion of probiotics and prebiotics increased protein availability and thus protein deposition (as a muscle protein) in the body of broilers [27]. For this reason, it is conceivable that *A. bilimbi* L. fruit filtrate (rich in organic acids), wheat bran (prebiotic source), and *S. cerevisiae* contributed to the increased protein biosynthesis, while reducing the protein breakdown, which was indicated by the higher pH values and WHC of meats.

The fatty acid profile is one of the most crucial factors determining the quality of broiler meats. In this study, the UFA-to-SFA ratio was notably higher in the breast meats of the treatment groups compared to those of the control. Del Puerto *et al*. [28] proposed the UFA-to-SFA ratio as a useful health indicator for meat, because a higher UFA-to-SFA ratio may protect consumers from hypercholesterolemia (a factor promoting atherosclerosis syndrome in humans). The feed additive proposed in this study may have this effect. A previous study reported that acidifiers reduced *de novo* synthesis of fatty acids in the liver, resulting in a lowered SFA content in broiler meat [10]. Similarly, Zhou *et al*. [29] reported that dietary supplementation of oligosaccharides lowered the SFA proportion in the breast muscle of broilers. Furthermore, Benamirouche *et al*. [30] reported decreased SFAs and increased UFAs in the breast meat of broilers fed with *S. cerevisiae*. Therefore, the proposed feed additive may contribute to lower *de novo* fatty acid synthesis and thus increase the UFA-to-SFA ratio. Our results show that the concentration of C20:3n-6 in the breast meat of broilers receiving 10 g/kg additive was lower than that of broilers receiving 2.5 and 5.0 g/kg additives. However, when comparing the treatments with the control, no such variation was observed.

Furthermore, the results reveal that L-glutamic acid was the most abundant of the amino acids in the breast meat (average 31.86 g/kg) followed by L-aspartate acid (19.94 g/kg) and L-lysine (19.35 g/kg). In addition, L-tyrosine was the least abundant amino acid (9.34 g/kg). The concentration of L-tyrosine in the breast meat of broilers receiving the additive at 5.0 g/kg was higher than that observed in control. A previous study reported that stress in chickens may be attributed to lowered tyrosine levels due to the increased dependence on the liver for synthesizing glucose, which is in part accomplished by the increased catabolism of glucogenic amino acids (including tyrosine) in the liver of chickens [31]. Therefore, we speculate that dietary administration of the proposed additive at 5 g/kg may alleviate stress in birds and thereby prevent tyrosine catabolism in the liver. However, this is not entirely clear because the tyrosine level in the breast meat of broilers receiving 10 g/kg additive was similar to that of the control. Finally, the concentrations of other glucogenic amino acids (i.e., threonine and glycine) did not change with the dietary administration of the proposed additive.

## Conclusion

Dietary supplementation of the mixture of *A. bilimbi* L. fruit filtrate, wheat bran, and *S. cerevisiae* improved the live BW and FCR of broilers. The feed additive also improved the physical and nutritional qualities of broiler breast meats, which is indicated by the increased UFA-to-SFA ratio in the sampled meats.

## Authors’ Contributions

SS: Designed the experiment, analyzed data, and drafted the manuscript. ARP and TY: Conducted *in vivo* experiment. TA: Designed the experiment and revised the manuscript. All authors read and approved the manuscript.
